# Supplementary material for: The genome sequence of the hoverfly, Orthonevra brevicornis (Loew, 1843) (Diptera: Syrphidae)
Source: Wellcome Open Res. 2025 Nov 5;10:616. [Version 1] doi: 10.12688/wellcomeopenres.25055.1 (PMC12743221; doi:10.12688/wellcomeopenres.25055.1)
Supplement: Peer review history

# Reviewer report: Reviewer response for version 1

**DOI:** 10.21956/wellcomeopenres.27621.r140330

**Published:** 2025-12-27

**Authors:** Jonathan Badger

**Affiliations:** 1 Laboratory of Integrative Cancer Immunology, National Cancer Institute, Bethesda, Maryland, USA

This is a straightforward genome assembly report and the background and methodology are well explained. I have only a few suggestions which are not critical and the authors can incorporate or not, as they choose. 

 1) Can you speculate on why there was such a difference in the assembly quality of the two haplotypes? 

 2) You explain that the the failure to obtain a Y chromosome may be that *O.* *brevicornis *males are X0, as some related species are known to be. While this is a reasonable hypothesis, is there any prior evidence that this is the case for *O.* *brevicornis?*

Are sufficient details of methods and materials provided to allow replication by others?

Yes

Is the rationale for creating the dataset(s) clearly described?

Yes

Are the datasets clearly presented in a useable and accessible format?

Yes

Are the protocols appropriate and is the work technically sound?

Yes

Reviewer Expertise:

I have been involved in genomics research for the past 25 years -- while I primarily work on microbial genomes and their interaction with their mammalian hosts, I have also worked on several insect genomics projects.

I confirm that I have read this submission and believe that I have an appropriate level of expertise to confirm that it is of an acceptable scientific standard.

# Reviewer report: Reviewer response for version 1

**DOI:** 10.21956/wellcomeopenres.27621.r140327

**Published:** 2025-12-24

**Authors:** Arun Arumugaperumal

**Affiliations:** 1 Department of Biotechnology, Rajalakshmi Engineering College, Thandalam, Chennai 602105, Tamil Nadu, India

The authors have presented the genome sequence of the hoverfly, *Orthonevra brevicornis*. An adult male has been used for sequencing. XY sex chromosomes were expected. But only the X chromosome was reported. The authors reason that the related species follow the X0 system in males. This is the first report of the high-quality genome sequence of this species. The authors have used PacBio long-read sequencing and Hi-C data to obtain a highly contiguous assembly. It is evident from the N50 value of 3.99 Mb. Further, they have resolved the haplotype and separately assembled the mitochondrial genome. The contigs have been scaffolded to create 6 chromosomal pseudomolecules. The assembly has reached an EBP metric of 6.C.Q64. The assembly and analysis have been carried out according to the standard procedures of the Darwin Tree of Life project. The genomic data is available in INSDC databases, and the accession links provided are active. The genome is yet to be annotated and is currently in the Ensembl annotation pipeline. Blobtoolkit GC coverage plot indicates that there are no contaminating reads. BUSCO percentage of 97.2 % indicates that most of the expected conserved genes are present in the assembly. The genome sequence will be useful in further molecular biology studies and studies concerning evolutionary relationships. The article can be indexed.

Are sufficient details of methods and materials provided to allow replication by others?

Yes

Is the rationale for creating the dataset(s) clearly described?

Yes

Are the datasets clearly presented in a useable and accessible format?

Yes

Are the protocols appropriate and is the work technically sound?

Yes

Reviewer Expertise:

Bioinformatics; Genomics

I confirm that I have read this submission and believe that I have an appropriate level of expertise to confirm that it is of an acceptable scientific standard.
